# Developing Rapid Antimicrobial Susceptibility Testing for Motile/Non-Motile Bacteria Treated with Antibiotics Covering Five Bactericidal Mechanisms on the Basis of Bead-Based Optical Diffusometry

**DOI:** 10.3390/bios10110181

**Published:** 2020-11-19

**Authors:** Yao-Tzu Yang, Jhih-Cheng Wang, Han-Sheng Chuang

**Affiliations:** 1 Department of Biomedical Engineering, National Cheng Kung University, Tainan 70101, Taiwan; p86074179@ncku.edu.tw; 2 Department of Urology, Chi-Mei Medical Center, Tainan 71004, Taiwan; tratadowang@stust.edu.tw; 3 Center for Micro/Nano Science and Technology, National Cheng Kung University, Tainan 70101, Taiwan

**Keywords:** antimicrobial susceptibility testing, Brownian motion, optical diffusometry, microbead, clinical sample, bacteria, antibiotics

## Abstract

Rapid antimicrobial susceptibility testing (AST) is an effective measure in the treatment of infections and the prevention of bacterial drug resistance. However, diverse antibiotic types and bacterial characteristics have formed complicated barriers to rapid diagnosis. To counteract these limitations, we investigated the interactions between antibiotic-treated bacteria and functionalized microbeads in optical diffusometry. The conjugation with bacteria increased the effective microbead complex size, thereby resulting in a temporal diffusivity change. The yielded data were sorted and analyzed to delineate a pattern for the prediction of antimicrobial susceptibility. The outcome showed that a completed rapid AST based on the trend of microbead diffusivity could provide results within 3 h (2 h measurement + 1 h computation). In this research, we studied four bacterial strains, including *Escherichia coli*, *Pseudomonas aeruginosa*, *Klebsiella pneumoniae*, and *Staphylococcus aureus*, and six antibiotics. Despite the different inhibitory effects caused by various antibiotics, similar trends in diffusivity alteration for all susceptible and resistant cases in the last 40 min of the 2-h measurement period were deduced. In addition, the AST results obtained using optical diffusometry showed good agreement with those acquired from the commercial instrument and conventional culture methods. Finally, we conducted a single-blinded clinical test, and the sensitivity, specificity, and accuracy of the system reached 92.9%, 91.4%, and 91.8%, respectively. Overall, the developed optical diffusometry showcased rapid AST with a small sample volume (20 μL) and low initial bacterial count (10^5^ CFU/mL). This technique provided a promising way to achieve early therapy against microbial diseases in the future.

## 1. Introduction

The rapid and precise determination of antibiotic sensitivity profile is pivotal in the successful treatment of an infection. As the pathogenic microbes turn noxious or resistant to drugs nowadays, the turnaround time to prescribe the correct antibiotics is a crucial matter to determine life or death. The current gold standard of antimicrobial susceptibility testing (AST) heavily relies on bacterial culture methods, such as broth microdilution [1] or disc diffusion [2], which require a long turnaround time (>24 h) [3,4] and a high initial bacterial count (at least 10^7^ CFU/mL) [3]. Currently, the application of commercial instruments (e.g., Biomerieux Vitek2 compact and BD Phoenix™ Automated Microbiology System) in clinical laboratory routines can save time by replacing labor-intensive work with automation. However, time-consuming bacterial culture remains inevitable. As a result, the current clinical practice still involves empirical antibiotic therapies [5], resulting in enhanced antibiotic resistance. Considerable approaches to accelerate the process, such as asynchronous bead rotation [6,7], dielectric electrophoresis [8], fluorescence intensity [9], Raman-enhanced spectroscopy [10,11], and single-cell imaging [12], have been developed to solve these shortcomings. Different mechanisms, like morphology, bacterial proliferator, fluorescence intensity, medium viscosity, and bacterial secretion, are used to determine the resistance of bacteria on antibiotics [13]. Although the processing time required for AST is substantially improved through these intensively developed methods, their potential uses in clinical applications remain limited because of expensive equipment, and highly demanding operating skills. 

**Figure 1:**
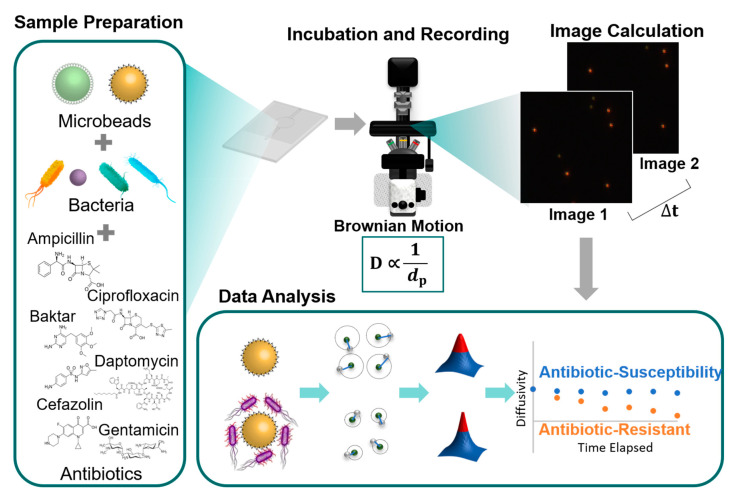
Schematic of the optical diffusometry for rapid antimicrobial susceptibility testing (AST). Four bacterial strains in combination with six antibiotics that cover five bactericidal mechanisms are investigated. The antimicrobial susceptibility is identified depending on the short trend of the temporal diffusivity change rather than the conventional long-term bacterial culture.

Alternatively, we have proposed an optical diffusometric platform composed of a fluorescent microscope and a digital camera in combination with functionalized microbeads to enable rapid AST [3,14]. Functionalized microbeads were coated with vancomycin to selectively capture bacteria. Instead of relying on external driving forces, a self-driving phenomenon, Brownian motion, which depends on ambient temperature, liquid viscosity, and microbead size [15,16], was used in the study. The relationship between the diffusivity and the ambient conditions is defined using the Stokes-Einstein equation [17]. Accordingly, the diffusivity varied in response to the bacterium–microbead interactions [18,19] because of the overall microbead complex size change [20,21]. Considering that the bacterial growth could be altered by antibiotics, the efficacy of the drug was measured from the temporal diffusivity change (Figure 1). The spatial cross-correlation algorithm was used to quantify the degree of Brownian motion from a series of consecutive microbead images [3,21,22]. Only an observation of diffusivity fluctuation in a short duration was needed to reveal the collective status of bacterial growth rather than a long bacterial culture. In comparison with our past work [3,14,23,24], this study focuses on the clinical practicability of the self-developed platform and comparisons with the conventional AST that rely on either manual bacterial culture or a commercial automated machine. In addition, a wide variety of diffusivity patterns resulting from interactions between four bacteria and six antibiotics were also investigated. Therefore, the new findings contribute to the realization of rapid AST from a practical perspective.

To further explore the capability of the optical diffusometry in rapid AST, four bacterial strains, i.e., Gram-positive/Gram-negative and motile/nonmotile bacteria, in combination with six antibiotics, were investigated in this study to ensure the clinical use of optical diffusometry. Common hospital-associated pathogens, including *Escherichia coli*, *Staphylococcus aureus*, *Pseudomonas aeruginosa*, and *Klebsiella pneumoniae*, were chosen to represent the Gram-positive/Gram-negative and the motile/nonmotile bacteria. Six antibiotics, ampicillin (AM), cefazolin (CZ), Baktar, ciprofloxacin (CIP), daptomycin (DAP), and gentamicin (GM), were used to represent five common bactericidal mechanisms. We conducted a trial to understand the interactions between the four bacteria and the six antibiotics, in which each bacterial strain was treated with six antibiotics separately. Collected responses and their effect on diffusivity were monitored in 2 h. Notably, the interactions between different bactericidal mechanisms and bacterial strains usually resulted in complicated behaviors in the first 80 min. Afterward, the diffusivity tended to decline with elapsed time when the bacteria showed resistance to the antibiotic because microbeads were bound to many bacteria and surrounded by rising liquid viscosity. Conversely, the trend of diffusivity levels halted as bacteria grew, and the rupture of bacteria caused by the activity of antibiotics, like DAP and GM, lead to a slight increase in diffusivity. Overall, only the slope of the temporal diffusivity changes in the last 40 min of the entire 2-h measurement period can account for the determination of AST. A preliminary assessment of 36 urine samples provided an optimal threshold (−0.0058 1/min in slope) defined by the receiver operating characteristic (ROC) curve for microbial susceptibility on our optical diffusometry. The single-blinded clinical test showed that the sensitivity, specificity, and accuracy reached 92.9%, 91.4%, and 91.8%, respectively. The proposed technique successfully demonstrated rapid AST in 3 h, including 2 h of measurement and 1 h of computation. Each measurement only required a small sample volume (20 µL) and a low initial bacterial count (10^5^ CFU/mL). This promising technique can enable early therapies against microbial diseases to save more lives in a timely fashion.

## 2. Materials and Methods

### 2.1. Reagents

Amine-modified fluorescent polystyrene (PS) microbeads (2 µm, L9529, orange, Sigma-Aldrich, St. Louis, MO, USA) were adjusted to a concentration of 2.8 × 10^7^ beads/mL [3] and functionalized using vancomycin hydrochloride (B1507, Biovision, Milpitas, CA) to selectively capture Gram-positive and Gram-negative bacteria [25,26]. Vancomycin possesses a similar structure to D-alanyl-D-alanine carboxypeptidase, which can form five hydrogen bonds with the D-Ala-D-Ala residues that exist on all bacterial cell walls [27]. Carboxylate-modified fluorescent PS microbeads (2 µm, L4530, yellow-green, Sigma-Aldrich, St. Louis, MO, USA) were used as a reference control by conjugating with anti-tumor necrosis factor (TNF) alpha monoclonal IgG (ab9348, Abcam, Cambridge, UK) to block bacteria or nonspecific bindings because TNF alpha is an inhibitor to pathogens [28]. 1-Ethyl-3-(3-dimethylaminopropyl)-carbodiimide (EDC, 1 mg/mL; A10807, Alfa Aesar, Haverhill, MA, USA) and *N*-hydroxysuccinimide (NHS, 1 mg/mL) were used to activate carboxylate groups for the formation of covalent bonds in the antibody conjugation. 2-(*N*-morpholino) ethanesulfonic acid (MES, 0.1 M, pH 5.5, M3671, Sigma-Aldrich, St. Louis, MO, USA) was used to adjust the pH to facilitate the conjugation. Tryptic soy broth (TSB; 211825, BD, Sparks, MD, USA), agar (HI-AGAR, Hispanagar, Burgos, Spain), and Lysogeny broth (LAB169, BioPioneer Tech., Taipei, Taiwan) were used for bacterial cultures. Phosphate-buffered saline (PBS; UR-PBS001, UniRegin Biotech, Taipei, Taiwan) was used as buffer solution for all assays. PBS Tween-20 (PBST) was prepared by mixing 0.01% Tween-20 (P5927, Sigma-Aldrich, St. Louis, MO, USA) with PBS to serve as a washing buffer. Saline (0.45% (*w/w*), SINTONG CO. LTD, Taoyuan, Taiwan) was used to adjust the bacterial concentration. 

### 2.2. Experimental Setup and Microchip Fabrication 

In this study, an inverted epifluorescent microscope (IX73, OLYMPUS, Tokyo, Japan) equipped with a green filter cube (U-MWIB3, OLYMPUS, Tokyo, Japan) and a 10× objective constituted the main body of the platform. A digital camera (FL3-U3-1382C-C8, Point Gray Research, Vancouver, Canada) was used to record consecutive microbead images at a frame rate of 10 Hz for 20 s for each measurement. Samples were incubated at 37 °C, whereas the laboratory temperature was maintained at 25 °C. The measurement uncertainty was estimated to be below 0.6% when the temperature variation was within ±1 °C [24]. Microbial sample droplets were confined in the microwells of a polymethylmethacrylate (PMMA) microchip and covered with a glass slide during the measurements. The microchip substrate was fabricated using a computer numerical control machine (EGX-400, Roland, Irvine, CA, USA). The PMMA microchip (Supplementary Material, Figure S1) had a channel width, channel depth, well diameter, and well depth of 1, 0.5, 6.5, and 0.28 mm, respectively. The microchip substrate and the glass slide were bonded with epoxy to seal the microwell and ensure no leakage and evaporation during measurement.

### 2.3. Functionalization of Probe Microbeads

For vancomycin-functionalized microbeads, 15 μL of 1 mg/mL EDC, 15 μL of 1 mg/mL NHS, and 50 μL of 0.1 M MES were mixed with 25 μL orange amine-modified PS suspension and 25 μL of 2 mg/mL vancomycin. The mixture was incubated in a shaker (800 rpm) and maintained at 4 °C for 4 h. Extra free suspending vancomycin was washed five times by centrifugation (10,000 rpm for 5 min) with PBST as the washing buffer and resuspended with MES before storage in the refrigerator (4 °C) overnight. For the reference microbeads, 25 μL green carboxylate-modified PS suspension was mixed with 15 μL EDC, 15 μL NHS, and 170 μL of 0.1 M MES buffer. Next, 5 μL of 2 mg/mL anti-TNF-α monoclonal IgG was added to the mixture to form the NH_3_-COOH bond in a shaker (800 rpm) at 4 °C for 4 h. Unconjugated antibodies were washed five times by centrifugation (10,000 rpm for 5 min) with the PBST buffer. The microbeads were functionalized completely and stored in a refrigerator (4 °C) overnight for later use. Readers who desire more detailed information concerning the characterizations of the vancomycin-coated microbeads and their interactions with bacteria can refer to our previous work [24].

### 2.4. Preparations of Bacteria and Antibiotics

Four bacterial strains (Supplementary Material, Figure S2), including *E. coli* (0.5–1 × 1.5–3 μm, ATCC 25922), *P. aeruginosa* (0.5–1 × 1.5–3 μm, ATCC 27853), *S. aureus* (*Φ* = 1–2 μm, ATCC 23360), and *K. pneumoniae* (0.5–1 × 1.5–3 μm, ATCC 700703), were purchased from American Type Culture Collection (Manassas, VA, USA) and used in this study. All bacteria were initially thawed at room temperature (25 °C) after retrieval from a freezer maintained at −30 °C. Subsequently, the bacteria were inoculated on tryptic soy agar and incubated at 37 °C for 12–16 h before use. When conducting AST, the initial bacterial count was adjusted to 0.53–0.6 McFarland with 0.45% (*w/w*) saline using a spectrophotometer (Vitek Densichek Analyzer BZA636869, BioMerieux, Marcy-l’Étoile, France). AM sodium salt (A9518, Sigma-Aldrich, St. Louis, MO, USA), Baktar (trimethoprim/sulfamethoxazole, 012528, SWISS PHARMACEUTICAL CO. LTD, Tainan, Taiwan), CZ sodium salt (C5020, Sigma-Aldrich, St. Louis, MO, USA), CIP (17850, Sigma-Aldrich, St. Louis, MO, USA), DAP (D2446, Sigma-Aldrich, St. Louis, MO, USA), and GM (G1264, Sigma-Aldrich, St. Louis, MO, USA) were used to inhibit bacterial growth. In addition to the standard testing in the hospital, a fraction of the same sample was measured using a commercial instrument (i.e., Vitek2) for comparison. The Vitek2 AST cards (AST-P638, AST-N329, GN ID [21341], and GP ID [21342]; Biomerieux, Marcy-l’Étoile, France) were selected in accordance with the identified bacterial strains. Sterilized Milli-Q water (Millipore, 18 MΩ) was used in all processes to avoid contamination. For the Vitek2 measurement, the 0.53–0.6 McFarland Gram-positive and Gram-negative bacterial solutions were diluted by 11- and 21-fold, respectively. The bacterial solutions and their corresponding AST cards were loaded into a cassette and installed in the machine for automated measurement. All procedures were performed following the Vitek2 protocol.

For the optical diffusometry, the initial bacterial count was also adjusted to 0.53–0.6 McFarland. The bacterial solution was mixed with Lysogeny broth, functionalized fluorescent PS microbeads, and antibiotics. The concentration of antibiotics followed the Vitek2 reports for these four bacteria to validate the performance between diffusometry and Vitek2. The mixture (20 μL) was pipetted into each microwell of the PMMA microchip and recorded using a series of images at a frame rate of 10 Hz for 20 s (Supplementary Material, Figure S3). A graph of temporal diffusivity changes over 2 h was depicted. The slope of the regression line in the last 40 min (i.e., the last three data points) was analyzed to identify the microbial susceptibility in response to a certain antibiotic. Unaffected bacteria (i.e., drug-resistant bacteria) tended to keep growing in the medium, thereby bringing down the diffusivity because of increased microbead complex size. Conversely, the inhibition of bacterial growth appeared to halt, resulting in a leveled or even slightly increased slope of diffusivity.

### 2.5. Preparation of Clinical Samples

For verification, the brightfield images of the clinical urine samples were always recorded before each measurement. During preparation, clinical samples were initially filtered with 5 μm filter paper to remove large fragments and subjected to centrifugation at 3000 rpm in a vial at 4 °C for 10 min to achieve a notable increase in the bacterial count (>10^4^ CFU/mL; Supplementary Material, Figure S9). The filtered samples were then mixed with functionalized fluorescent PS microbeads and the Clinical and Laboratory Standards Institute-guided concentrations of antibiotics for *E. coli* in Eppendorf tubes. After thorough agitation, 20 μL mixed liquid was transferred to each microwell of the PMMA microchip and incubated at 37 °C for 2 h. Data were measured every 20 min during incubation. The final result could be reported after about 1 h of computation, making the turnaround time around 3 h. Even with the sample pretreatment, the overall time needed from sampling to reporting was 4 h.

For Vitek2, however, the samples were inoculated on tryptic soy agar and incubated in an oven at 37 °C overnight. When conducting AST, the initial bacterial count was carefully adjusted to 0.53–0.6 McFarland with 0.45% saline by using a spectrophotometer. The unknown samples were examined for their strains with ID cards (4–13 h). Subsequently, the bacterial solution and the AST cards were loaded into a cassette and inserted into the Vitek2 machine for automated measurement. The AST results could be reported after another 10 h of machine operation. Unlike the optical diffusometry, the bacterial culture was an inevitable step in the operation, resulting in a lengthy turnaround time (>24 h, Supplementary Material, Figure S4). A conventional AST assay was also conducted in the hospital to provide a reference standard for comparison. However, the result was usually obtained after a week.

### 2.6. Mechanisms of Bacterial Inhibition from Antibiotics

Four representative bacteria were investigated to reveal how the temporal diffusivity may change in response to the bacterial growth. The bacteria were classified in terms of their motility and Gram type (Supplementary Material, Figure S2). *E. coli*, *P. aeruginosa*, and *K. pneumoniae* were Gram-negative. These bacteria are rod-like microbes [29,30,31] that cause many commonly infectious diseases, such as urinary tract infections (UTIs) [31,32]. *E. coli* and *P. aeruginosa* are motile with energy imparted by their flagella. Conversely, *S. aureus* is a Gram-positive round-like microbe that can be frequently found in the upper respiratory tract and on the skin [30]. Methicillin-resistant *S. aureus*, one of the drug-resistant superbugs evolving from this strain, is a major threat to modern human society. Notably, Gram-positive and motile microbes were unusually reported in modern hospitals and were not included in this study.

**Figure 2:**
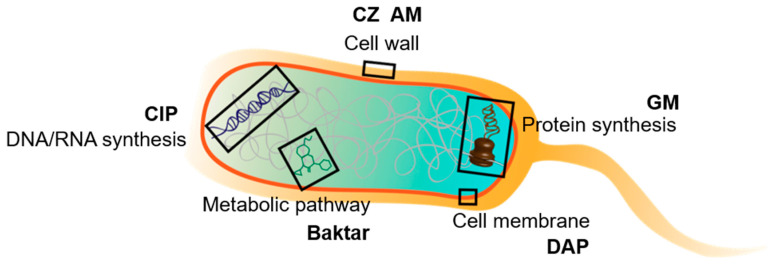
Schematic of the major bactericidal mechanisms of the six selected antibiotics.

Six antibiotics, which covered five major bactericidal mechanisms (i.e., inhibition of cell wall synthesis, protein synthesis, membrane synthesis, metabolic pathway, and DNA/RNA synthesis; Figure 2) were used to investigate their influences on the four selected bacteria. The mechanism of each antibiotics and the AST results can be found at Table S1. AM and CZ were used to treat infections from Gram-positive and Gram-negative bacteria [33] by inhibiting their cell wall synthesis [33,34]. The morphology of pre-existing bacteria was unaffected, but all newly produced bacteria grew abnormally in shape (i.e., longer) and easily ruptured during osmotic changes [35]. These drugs can induce bacteria to grow as large cells with less frequent cell divisions by affecting the crosslinking of the bacterial cell wall. Baktar consists of a combination of two antifolate agents, namely trimethoprim and sulfamethoxazole [36]. These agents inhibit successive steps in the folate synthesis pathway [37], causing two effects, namely elongation and membrane disruption [38] in cells. CIP is a second-generation fluoroquinolone with a broad spectrum of activity that usually results in the death of the bacteria [39] due to the inhibition of DNA synthesis [40]. CIP causes cell elongation and filaments or ovoid cells at different concentrations. Past research on their effects on *E. coli* show that filamentation and vacuolation appear after approximately 1 h of exposure to CIP but rupture and deflate after 2 h [41]. Most of the unusual and bizarre morphologies caused by DAP led to bacterial shrinkage, resulting in increased diffusivity. DAP is a lipopeptide antibiotic used in the treatment of systemic and life-threatening infections caused only by Gram-positive organisms [42]. DAP inhibits protein, DNA, and RNA syntheses by disrupting the bacterial cell membrane permeability, which can eventually cause cell death [43]. The antibiotic-induced morphological changes include antler-like protrusions, minicells, elongated linear cells, blebs, and deep, web-like fissures [44]. In addition, GM inhibits microbes by binding the 30S subunit of the bacterial ribosome, negatively affecting protein synthesis [45]. GM has profound effects on the morphological structure of bacteria, of which bacterial lysis is the most commonly observed [46].

### 2.7. Determination of the Optimal Threshold for Rapid AST

**Figure 3:**
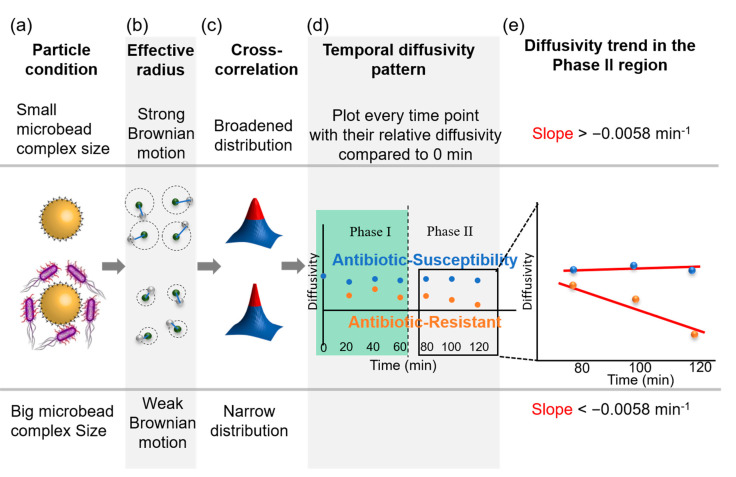
Relative diffusivity of microbeads at two different conditions and determination of threshold slope. (**a**) Particle condition of susceptibility and resistant groups. (**b**) Effective radius of two conditions causing the different intensities of Brownian motion. (**c**) Particle images wherein spatial cross-correlation, a statistical algorithm, is adopted. (**d**) Relative diffusivity every 20 min. (**e**) Regression line slope of the last three points.

The comprehensive AST evaluation of optical diffusometry requires the consistent monitoring of bacterial activity for 2 h and image computation for 1 h. Bacteria, antibiotics, and the functionalized microbeads were incubated in the microchip simultaneously. As bacteria grew during the measurement, the microbead complex (i.e., bacteria-microbead composite) changed in volume through the capture of the bacteria (Figure 3a). In general, the diffusivity decreased with increased microbead complex size (Figure 3b), but might show erratic behavior when more factors, such as bacterial motility and morphological changes, are considered. A statistical algorithm, termed spatial cross-correlation, was used to produce a representative analysis (Figure 3c). The theoretical model in the previous section suggested that a slope of temporal diffusivity change in the phase II region exceeding a critical threshold revealed a highly positive correlation with the efficacy of the antibiotics for inhibiting bacteria (Figure 3d). Notably, the determination was performed in accordance with the slope of diffusivity change in phase II because the antibiotics required time (>80 min) to take effect on bacteria (Figure 3e).

## 3. Results and Discussion

### 3.1. Empirical AST Threshold Derived from Clinical Samples

A total of 36 clinical samples (Supplementary Material, Figure S5a) were collected from patients with confirmed UTIs at the Chi-Mei Medical Center (IRB agreement #10312-004) and subjected to optical diffusometry to get an eligible threshold in clinical practice. Microbead images were recorded in the brightfield and fluorescence modes. The brightfield mode was used to demonstrate the proliferation of bacteria and their interactions with microbeads while the fluorescence mode (Supplementary Material, Video S1) was to provide pure microbead images for the Brownian motion analysis. Notably, the samples were initially processed with filtration and enrichment before measurement. The slopes of all patients’ diffusivity changes in phase II were calculated, and the ROC curve was plotted (Supplementary Material, Figure S5b). An optimal threshold, −0.0058 1/min, was determined by the shortcut from the ROC curve to the ideal point, which was located in the upper left corner. With this estimated threshold, the sensitivity, specificity, and accuracy of this technique could eventually reach 87.1%, 80%, and 86.1%, respectively.

### 3.2. Diffusivity Changes in Response to Interactions between Bacteria and Antibiotics

**Figure 4:**
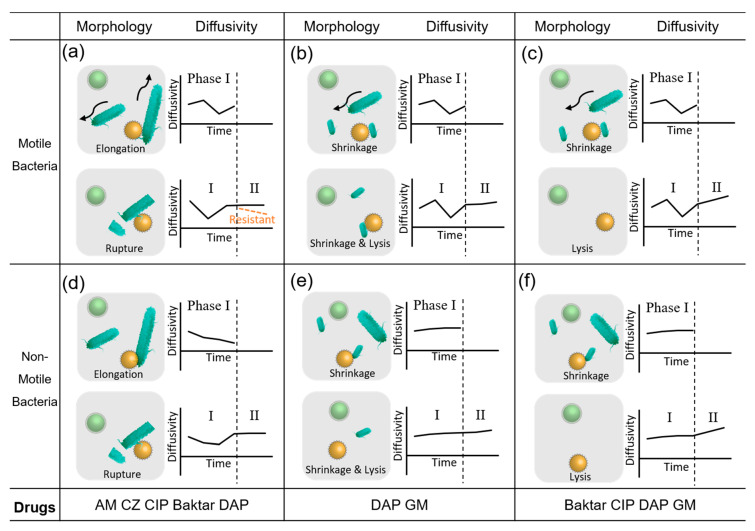
Organized diffusivity patterns based on the morphology of (**a**–**c**) motile and (**d**–**f**) nonmotile bacteria treated with the six selected antibiotics. The last column indicates the possible antibiotics in this research. The gray square shows microbeads and changing bacterial morphology at Phases I and II. Each plot shows the possible diffusivity patterns.

In accordance with the abovementioned bacterial characteristics (Figure 2) and the potential diffusivity variations due to their morphological changes, the interactions between bacteria and effective antibiotics were divided into six diffusive patterns (Figure 4a–f). In the motile bacterial group, the diffusive patterns for the two morphological changes (i.e., elongation and shrinkage) showed fluctuating behavior in phase I (first 80 min of the total 2-h measurement) because the major dominant factor was from the extra energy induced by bacterial motility (Figure 4a–c). A clear peak can be even observed if the bacterial motility outperforms the morphological changes [14]. At measurement in the phase II region (last 40 min of the total 2-h measurement), the diffusive pattern tends to mitigate or level off because of bacterial rupture. Notably, the timing was empirically determined by the experimental observations. For some slow-reproducing bacteria, such as *Mycobacterium tuberculosis*, however, the timing will need to be redefined. Conversely, the drug-resistant bacteria showed slope values lower than the threshold (Figure 4a). The diffusive pattern increased as the bacteria shrank or were lysed by antibiotics (Figure 4b,c). In the nonmotile bacterial group, fluctuation seemed to be milder than their motile counterparts because of the lack of external motility of bacteria. For those that showed elongation in phase I (Figure 4d), the diffusivity exhibited a declining trend. By contrast, the occurrences of shrinkage and lysis in phase I led to a slightly increased slope in diffusivity (Figure 4e,f). When bacteria ruptured after the antibiotic took effect in phase II, the diffusive pattern increased (Figure 4d). However, if the bacteria shrank or were lysed, the diffusive patterns slightly increased in response to the decrease in the microbead complex volume (Figure 4e,f). Notably, the diffusive patterns for the morphological changes showed a monotonically declining slope in phase II when bacteria expressed obvious resistance to the antibiotics. Considering the four bacteria usually reproduce themselves every 20 min to 2 h [47,48], the morphological changes in the bacteria, i.e., elongation/rupture/lysis, were detected within the 2-h measurement. Overall, we observed that fast-reproducing bacteria (<2 h) were more likely to result in a similar diffusivity trend in the phase II region.

### 3.3. Validations of the Optical Diffusometry

Four standard ATCC bacterial strains were measured on the optical diffusometry platform. Each bacterium was initially paired with the six selected antibiotics at their minimum inhibitory concentrations (MICs) suggested by the outcomes from a commercial AST machine (Vitek2, Biomerieux). However, *K. pneumoniae* expressed strong drug-resistant behavior, resulting in only MICs of Baktar and CIP being found. Concentrations above and below the MIC for some bacteria was conducted in our past studies [3,14]. All low antibiotic concentrations (<MIC) showed lower slopes than the threshold in phase II and vice versa. The control group was not treated with any antibiotic. All measured data were compared with those measured from Vitek2. For simplicity, the bacteria against antibiotics in the study were only determined to be either susceptible (S) or resistant (R) because our system was incapable of determining an intermediate dosage (I). Unlike the susceptible dosage or resistant dosage, the intermediate dosage has a likelihood of therapeutic uncertainty [49]. Considering safety in clinical use, a higher dose is usually suggested (by the European Committee on Antimicrobial Susceptibility Testing, EUCAST) when an intermediate outcome is obtained in an AST test.

**Figure 5:**
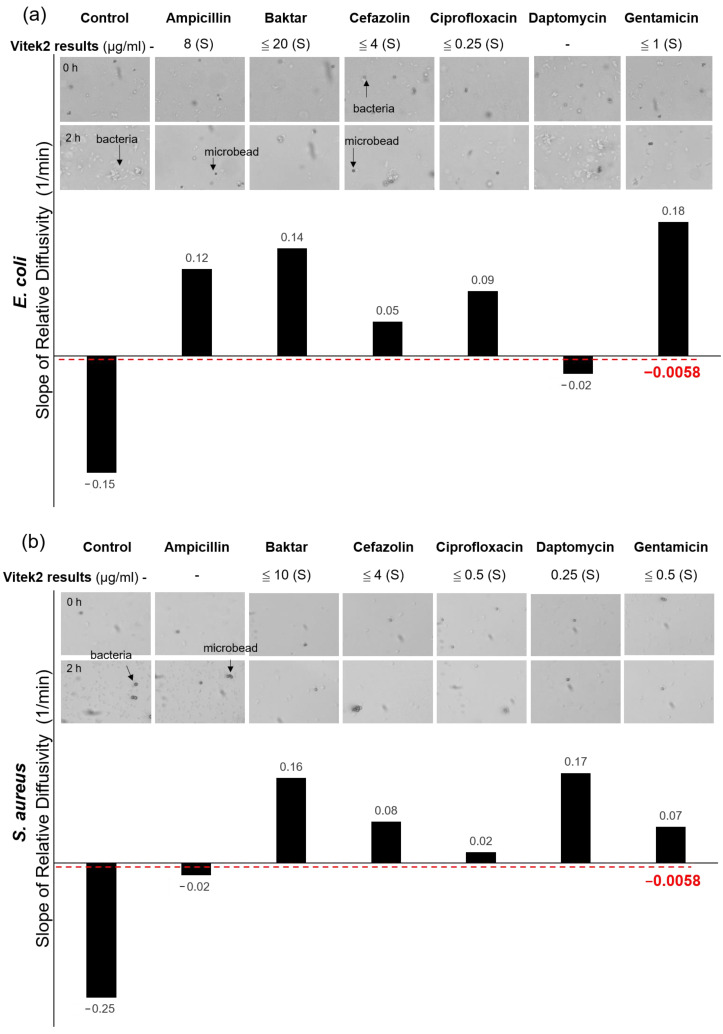
Comparisons between our optical diffusometry and the Vitek2 with the ATCC *E. coli* (**a**) and *S. aureus* (**b**) strain. The Vitek2 results and their corresponding minimal inhibitory concentrations (MICs) of antibiotics are annotated below the names of bacteria. Brightfield images (10× objective) show the actual bacterial growth at the beginning and after 2 h of incubation. The bright and rod-like shapes are bacteria, whereas the dark grey circles are the microbeads. The bars show the diffusometric AST outcomes in slope. Positive and negative values represent bacterial susceptibility and resistance. The dotted red line stands for the threshold for the AST discrimination on the optical diffusometry. The symbol “-” in the Vitek2 results indicates no data measured from the machine.

**Figure 6:**
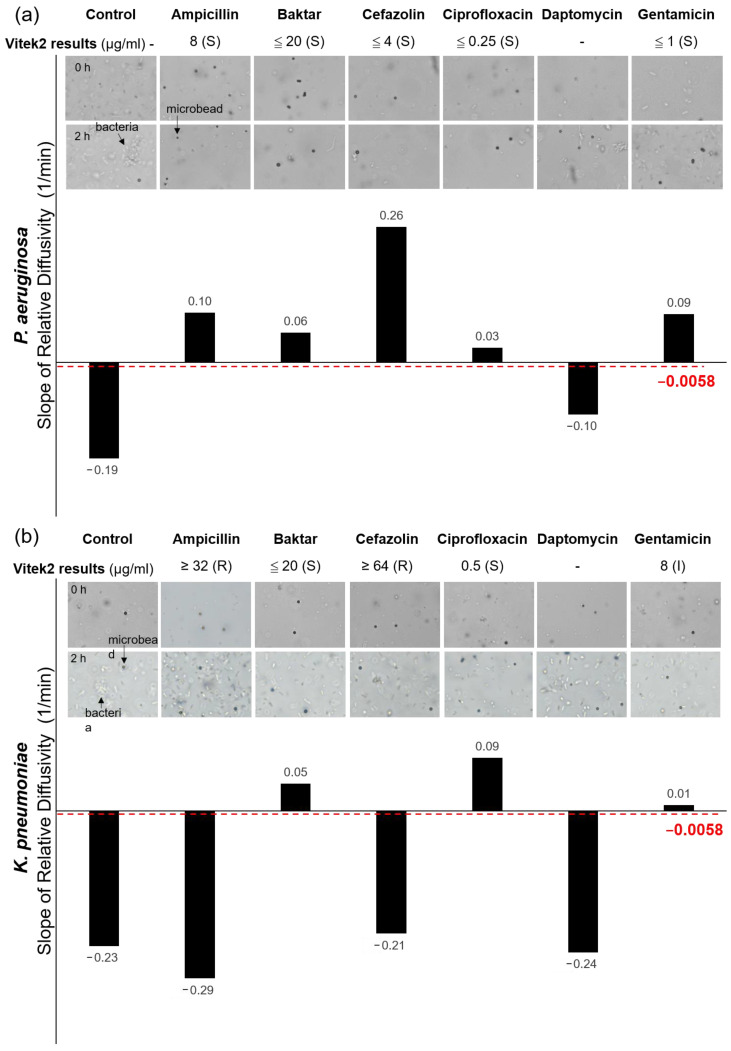
Comparisons between our optical diffusometry and the Vitek2 with the ATCC *P. aeruginosa* (**a**) and *K. pneumoniae* (**b**) strain. The Vitek2 results and their corresponding MICs of antibiotics are annotated below the names of bacteria. Brightfield images (10× objective) show the actual bacterial growth at the beginning and after 2 h of incubation. The bright and rod-like shapes are bacteria, whereas the dark grey circles are the microbeads. The bars show the diffusometric AST outcomes in slope. Positive and negative values represent bacterial susceptibility and resistance. The dotted red line stands for the threshold for the AST discrimination on the optical diffusometry. The symbol “-” in the Vitek2 results indicates no data measured from the machine.

In the *E. coli* group (Figure 5a), only the slopes of the control and the DAP were lower than −0.0058 1/min, whereas the other antibiotics exhibited slopes higher than the threshold. The outcomes explicitly indicated that 0.25 μg/mL DAP failed to inhibit the growth of *E. coli*. The data were in good agreement with those measured from the Vitek2. In the *S. aureus* group (Figure 5b), only the slopes of the control and the AM were lower than −0.0058 1/min among all the tested antibiotics, indicating that the *S. aureus* strain was resistant to 8 μg/mL AM. Notably, DAP and AM were not seen in the measurements of Vitek2 because the AST cards used for *E. coli* and *S. aureus* did not include both antibiotics. However, the brightfield images of the bacterial growth during the 2-h period were recorded to provide direct evidence to verify the measurements. Evidently, both cases showed substantial bacterial growth in 2 h. In the *P. aeruginosa* group (Figure 6a), the control and the DAP showed negative responses (resistant), whereas Baktar, CZ, CIP, and GM showed positive responses (susceptible). *P. aeruginosa* has strong mobility, which may interfere with the Brownian motion (21, 41). The results did not seriously deviate from their Vitek2 counterparts. In the *K. pneumoniae* group (Figure 6b), the bacteria expressed strong drug resistance under the treatments of AM, CZ, and DAP but were susceptible in CIP and Baktar. The results were consistent for the optical diffusometry and Vitek2. Notably, the bacteria treated with GM in Vitek2 showed an intermediate outcome. Considering the uncertainty of intermediate dosage, as used in the human body, it is usually recognized as resistant in most clinical practice. Although the result appeared susceptible in our optical diffusometry measurement, the weak positive response appeared insufficient to support the efficacy of GM. The result confirmed the uncertainty of 4 μg/mL GM on the *K. pneumoniae*.

The detailed diffusivity data of the four bacteria with different antibiotics recorded every 20 min within 120 min are shown in the Supplementary Material (Figures S6 and S7). In general, different antibiotics were capable of leading distinct morphologies depending on the biological mechanisms. However, some minor differences were still observed, even with the same antibiotic treatment and bacterial strain. Nevertheless, the overall trends of temporal diffusivity changes in the last 40 min in response to susceptibility and resistance were still quite similar because the dominant factors that affected the Brownian motion relied mainly on the bacterial count in liquid medium, liquid viscosity, bacterial morphology (elongation/rupture/lysis), and bacterial motility. Eventually, our technique successfully demonstrated the effects of four bacteria treated with six antibiotics at their MICs. Through the validation by Vitek2, our optical diffusometry was rapid in the AST reports and yielded 100% sensitivity, 90.9% specificity, and 96.4% accuracy.

### 3.4. Evaluations of the Clinical Samples

Clinical urine samples were obtained from seven patients with UTIs in the Chi-Mei Medical Center (IRB agreement #10312-004). On the basis of the optimized optical diffusometry, a single-blinded test was conducted. Each sample was divided into three copies, and their AST data were separately measured from the hospital, the Vitek2, and the optical diffusometry (Table 1; Supplementary Material, Figures S10 and S11; Table S2). In the control column, the bacterial strain was identified in the hospital and in the Vitek2. For simplicity and to save time, the bacterial identity was not determined in the optical diffusometry. As a result, the outcome of the optical diffusometry only reported the existence of pathogens and the efficacy of antibiotics. When there were no bacteria or the bacterial count was too low to be detected, there would be nearly no diffusivity changes over the 2-h measurement. In samples 1, 2, and 4, the bacterial counts were too low to be detected in all three methods. In sample 3, the results from the hospital and the Vitek2 were generally the same, except for the bacteria treated with CZ. As the results from the Vitek2 and our optical diffusometry were compared, inconsistent results were observed in those bacteria treated with Baktar, CIP, and GM. The inconsistency was likely attributed to the abnormally high initial bacterial counts, which caused massive dead cell debris that interfered with the Brownian motion (Supplementary Material, Figure S8). Comparatively, all other AST results in samples 5–7 were in good agreement. Although sample 6 had notably low initial bacterial counts that obstructed the conventional culture, the low bacterial growths were still successfully detected by the optical diffusometry and the Vitek2. Considering that optical diffusometry and the Vitek2 were operated with bacteria growing in liquid forms (disk diffusion method in hospital is solid forms), the data of the Vitek2 were more comparable with that of the optical diffusometry. By taking Vitek2 as a standard, the sensitivity, specificity, and accuracy of the single-blinded test on the optical diffusometry reached 92.9%, 91.4%, and 91.8%, respectively (Supplementary Material Table S2). Although only two bacterial strains, *K. pneumoniae* and *E. coli*, were found in the samples, they still belonged to the four selected bacteria. This clinical test proved that the proposed technique successfully provided comparable data for the commercial benchtop, Vitek2, and conventional AST in a short turnaround time (3 h).

## 4. Conclusions

To achieve rapid AST for four bacteria treated with six antibiotics, we developed a diffusometry-enabled biosensing platform. The interactions between bacteria and the antibiotics were monitored by functionalized microbeads because Brownian motion was inversely proportional to the microbead complex size. The trend of diffusivity change reflected the information about the antimicrobial susceptibility of bacteria bound to the functionalized microbeads. Using vancomycin-functionalized microbeads as probes, we examined four kinds of bacteria, including *E. coli*, *P. aeruginosa*, *S. aureus*, and *K. pneumoniae*, which covered the domains of Gram-positive/Gram-negative and motile/nonmotile, in combination with six antibiotics associated with five bactericidal mechanisms. The outcomes obtained from Vitek2 and optical diffusometry were mostly in good agreement. The comparison validated our belief that the proposed optical diffusometry was capable of conducting AST with a broad spectrum of bacteria treated with various antibiotics. Because of the high sensitivity of bead-based optical diffusometry, only the diffusivity change in the last 40 min of the entire 2 h measurement was enough to draw results for the AST identification. An AST evaluation with 36 clinical samples suggested that the threshold was −0.0058 1/min. Accordingly, the “S” response occurred when the measured diffusivity change exceeded the threshold and vice versa. Compared with a well-adopted AST machine (i.e., Vitek2), our optical diffusometry appeared to show highly consistent results. Eventually, a single-blinded test with seven clinical samples was conducted to verify the practicability of the technique. The sensitivity, specificity, and accuracy reached 92.9%, 91.4%, and 91.8%, respectively. In conclusion, this study showcased a proposed technique that featured rapid AST (~3 h = 2-h measurement + 1-h computation), a small sample volume (20 µL), and a low initial bacterial count (10^5^ CFU/mL). The technique provided a promising alternative that will enable us to achieve rapid therapies against severe infectious diseases.

## Figures and Tables

**Table 1:** Comparisons of clinical samples measured from the hospital (H), Vitek2 (V), and the optical diffusometry (O) under the single-blinded test.

#		Control	Ampicillin	Baktar	Cefazolin	Ciprofloxacin	Daptomycin	Gentamicin
1	H	<(GNB)*	-***	-	-	-	-	-
	V	No colony	-	-	-	-	-	-
	O	No bacteria	-	-	-	-	-	-
2	H	<(GNB)	-	-	-	-	-	-
	V	No colony	-	-	-	-	-	-
	O	No bacteria	-	-	-	-	-	-
3	H	*Klebsiella pneumoniae*	R	S	R	S	-	S
	V	*Klebsiella pneumoniae*	I	S	S	S	-	S
	O	√**	R	R	S	R	S	R
4	H	0 colony	-	-	-	-	-	-
	V	No colony	-	-	-	-	-	-
	O	No bacteria	-	-	-	-	-	-
5	H	*Escherichia coli*	S	S	S	S	-	S
	V	*Escherichia coli*	S	S	S	S	-	S
	O	√	S	S	S	S		S
6	H	<(GNB)	-	-	-	-	-	-
	V	Low Discrimination (*Escherichia coli*)	R	R	R	R	-	S
	O	√	R	R	R	R	R	S
7	H	*Escherichia coli*	R	S	I	S	-	S
	V	*Escherichia coli*	R	S	S	S	-	S
	O	√	R	S	S	S	R	S

* Gram negative bacillus, <10^4^ CFU/mL, no AST report. ** “√” denotes bacteria detected. *** “-” denotes no data measured.

## Supplementary Materials

The following are available online at https://www.mdpi.com/2079-6374/10/11/181/s1, Figure S1: Illustrative dimensions of the microchip; Figure S2: Four ATCC bacterial strains and their classifications in terms of Gram type and motility; Figure S3: Preparations of bacteria and antibiotics for the optical diffusometry and Vitek2 measurements; Figure S4: Timelines of the optical diffusometry, Vitek2, and the conventional culture in a hospital; Figure S5a: Detailed information of the 36 clinical samples; Figure S5b: ROC curve of the 36 clinical samples; Figure S6: Temporal diffusivity changes of *S. aureus* and *E. coli* treated with six antibiotics in 2 h; Figure S7: Temporal diffusivity changes of *K. pneumoniae* and *P. aeruginosa* treated with six antibiotics in 2 h; Figure S8: Bright-field images of bacterial growth of clinical sample 3 shown in Table 1; Figure S9: Bacterial count enrichment. Figure S10: Diffusivity patterns and bright-field images recorded at the beginning and after 2-h incubation of samples 3 and 5; Figure S11: Diffusivity patterns and bright-field images recorded at the beginning and after 2-h incubation of samples 6 and 7; Table S1: Bactericidal mechanisms of the six selected antibiotics and their inhibitory effects on the four bacteria; Table S2: Classifications of positive and negative groups for the 7 clinical results Video S1: Particle Brownian motion recorded under the fluorescent mode, https://drive.google.com/file/d/1pBLqqAh_RItEdSuXOamLPu1QNRXasU-W/view?usp=sharing.
