# Supplementary material for: Developing Rapid Antimicrobial Susceptibility Testing for Motile/Non-Motile Bacteria Treated with Antibiotics Covering Five Bactericidal Mechanisms on the Basis of Bead-Based Optical Diffusometry
Source: Biosensors (Basel). 2020 Nov 19;10(11):181. doi: 10.3390/bios10110181 (PMC7699397; doi:10.3390/bios10110181)

Developing Rapid Antimicrobial Susceptibility Testing for Motile/Non-motile Bacteria Treated with Antibiotics Covering Five Bactericidal Mechanisms on the Basis of Bead-Based Optical Diffusometry

**Yao-Tzu Yanga, Jhih-Cheng Wang^b^, and Han-Sheng Chuanga, c, ***

^a^ Department of Biomedical Engineering, National Cheng Kung University, Taiwan

^b^ Department of Urology, Chi-Mei Medical Center, Taiwan

^c^ Center for Micro/Nano Science and Technology, National Cheng Kung University, Taiwan

*[oswaldchuang@mail.ncku.edu.tw](mailto:oswaldchuang@mail.ncku.edu.tw)

Supporting Information


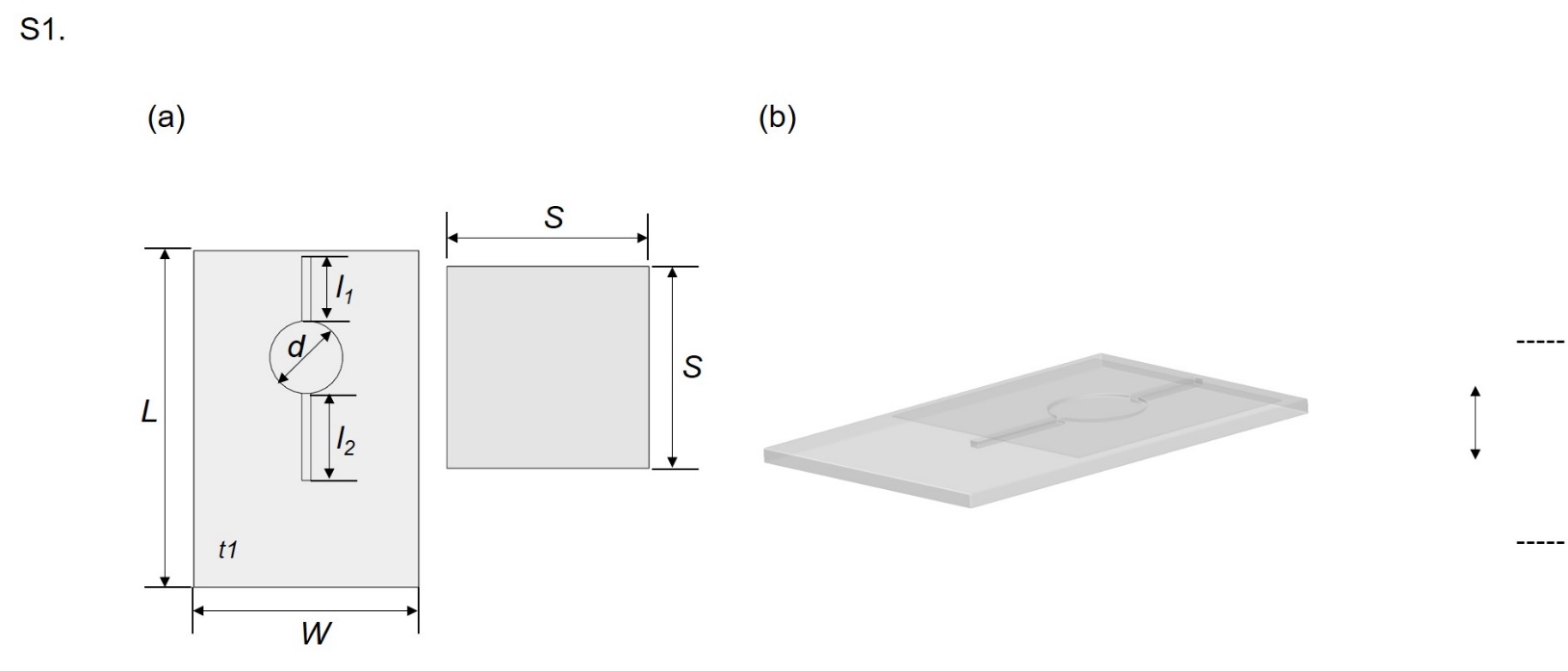
**Figure S1.** Illustrative dimensions of the microchip. (a) Bulk PMMA substrate (left) and cover glass (right). The major dimensions of the PMMA are L = 30 mm, W = 20 mm, d = 6.5 mm, l_1_ = 5.75 mm, l_2_ = 7.75 mm. Depth of the circle is 0.28 mm. Depth of the channel is 0.5 mm. Thickness of the PMMA substrate is t1 = 1 mm. The side length of the cover glass is S = 18 mm. (b) A perspective view of the assembled microchips.


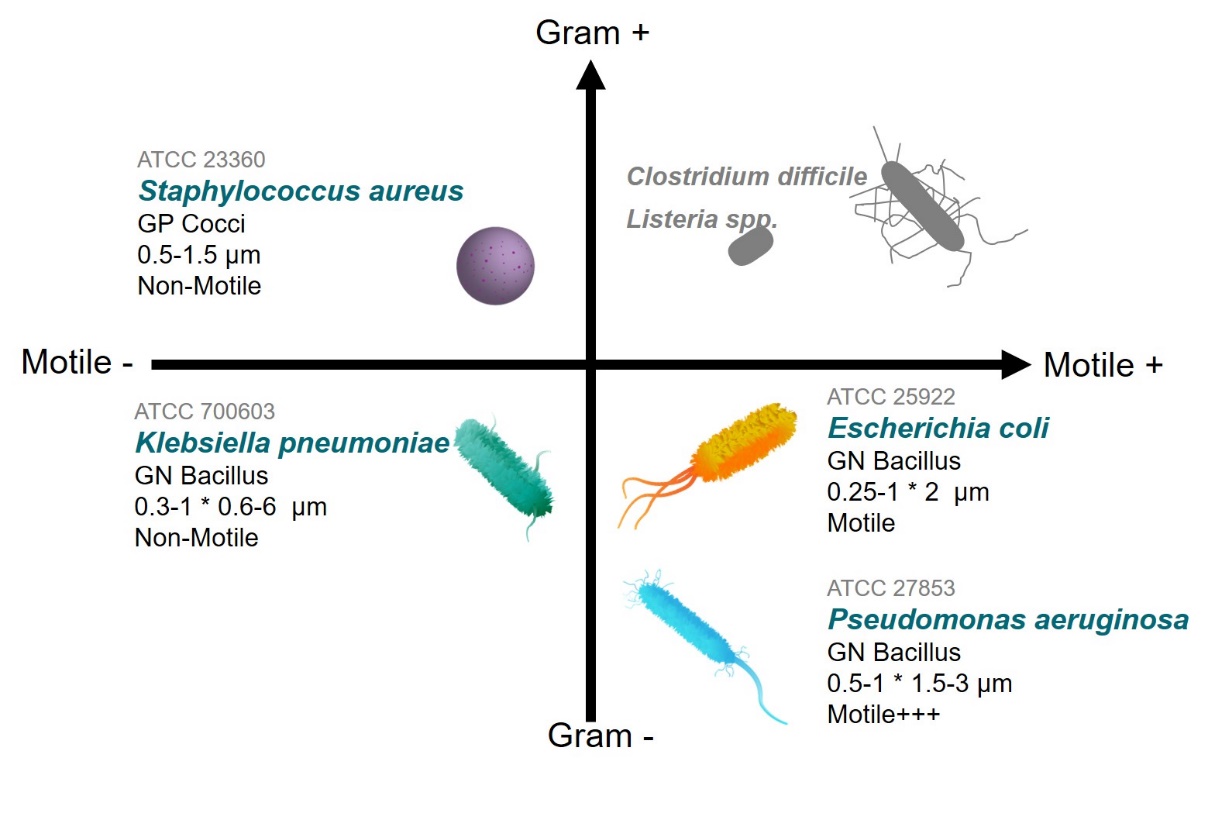


**Figure S2.** Four ATCC bacterial strains and their classifications in terms of Gram type and motility. *S. aureus* belongs to the 2^nd^ quadrant, which is defined a non-motile, Gram-positive, and round-shaped (cocci) bacterium. *K. pneumoniae* belongs to the 3^rd^ quadrant, which is defined a non-motile, Gram-negative, and rod-shaped (bacillus) bacterium. *E. coli* and *P. aeruginosa* belong to the 4^th^ quadrant, which are defined as motile, Gram-negative, and rod-shaped bacteria.


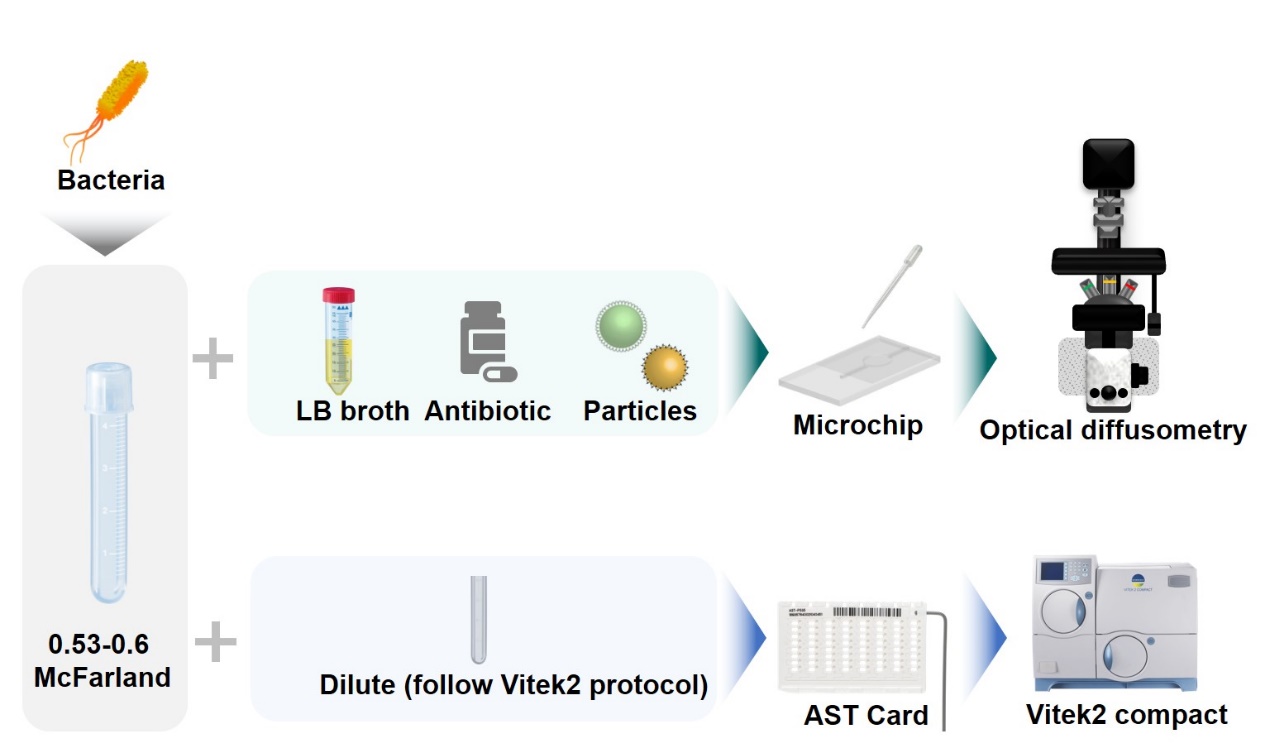


**Figure S3.** Preparations of bacteria and antibiotics for the optical diffusometry (top row) and Vitek2 measurements (bottom row).


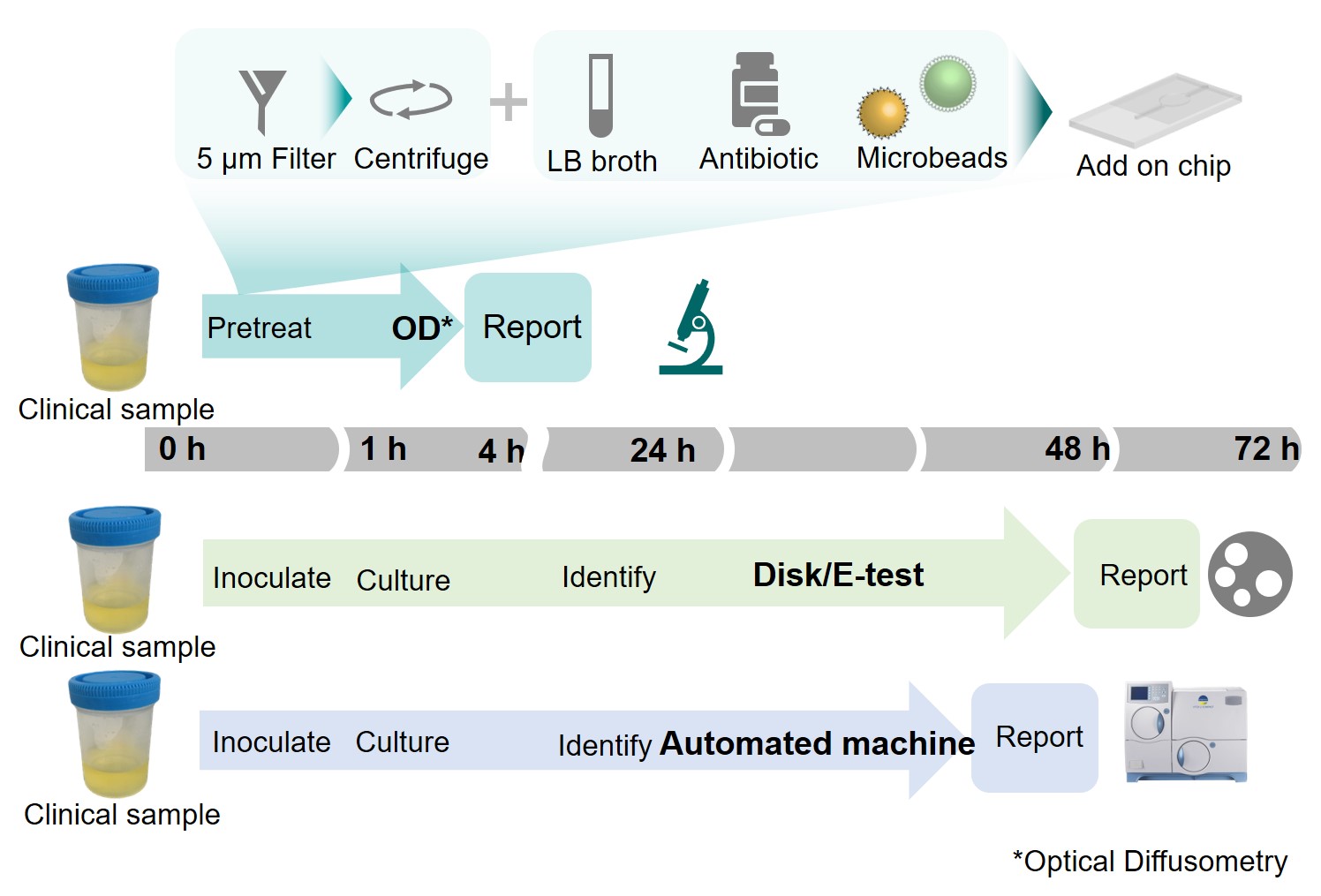


**Figure S4.** From sample to report timelines of the optical diffusometry, Vitek2, and the conventional culture in a hospital.


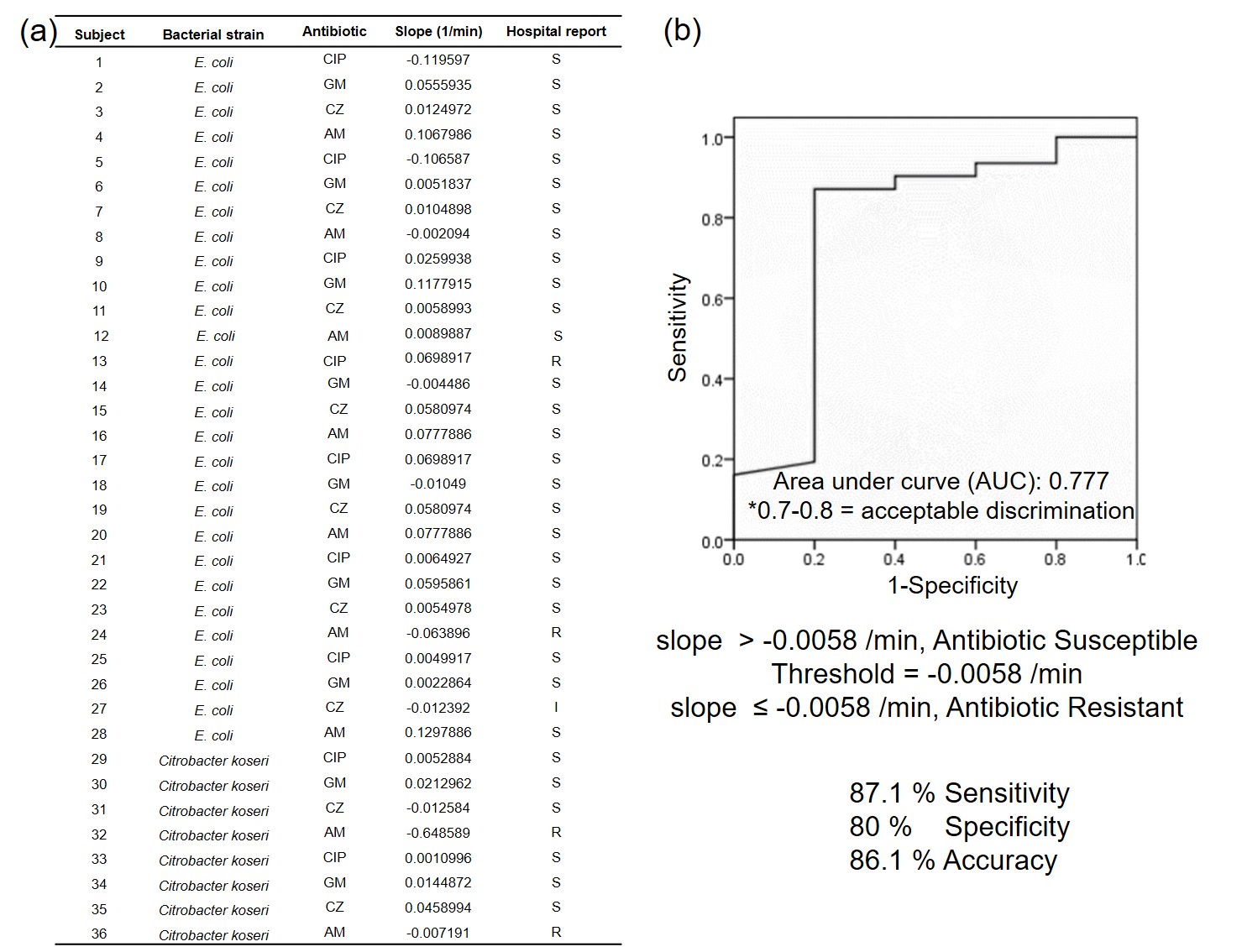


**Figure S5.** (a) Detailed information of the 36 clinical samples. (b) ROC curve of the 36 clinical samples. The optimal threshold is determined to be -0.0059 1/min. The sensitivity, specificity, and accuracy of the optical diffusometry based on the clinical samples are 87.1%, 80%, and 86.1%, respectively. All antibiotics were tested at the MIC for the respective bacterium.


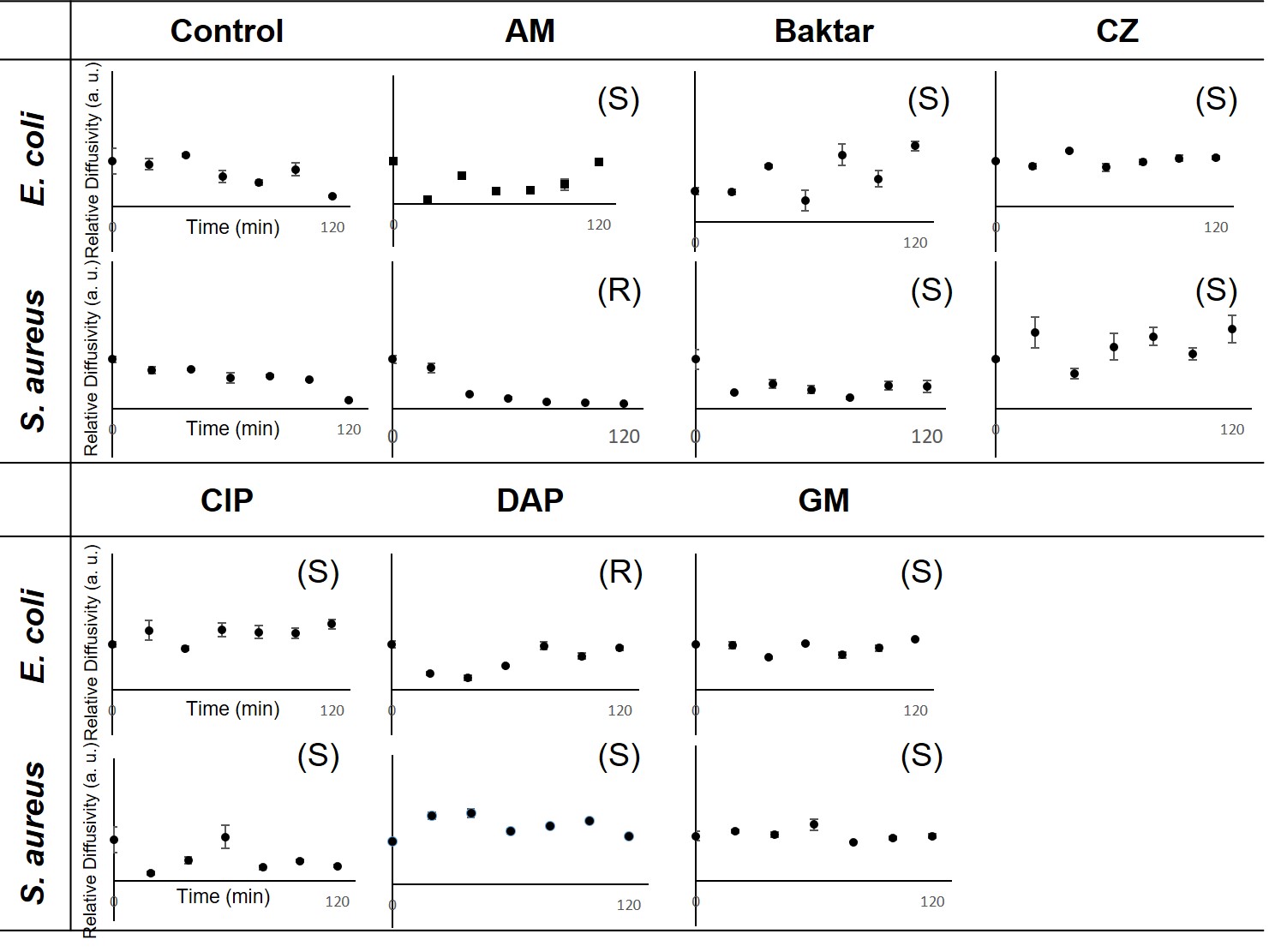


**Figure S6.** Temporal diffusivity changes of *S. aureus* and *E. coli* treated with six antibiotics in 2 h. The data were measured every 20 min (n=5). “S” means susceptible while “R” means resistant. All antibiotics were tested at the MIC for the respective bacterium.


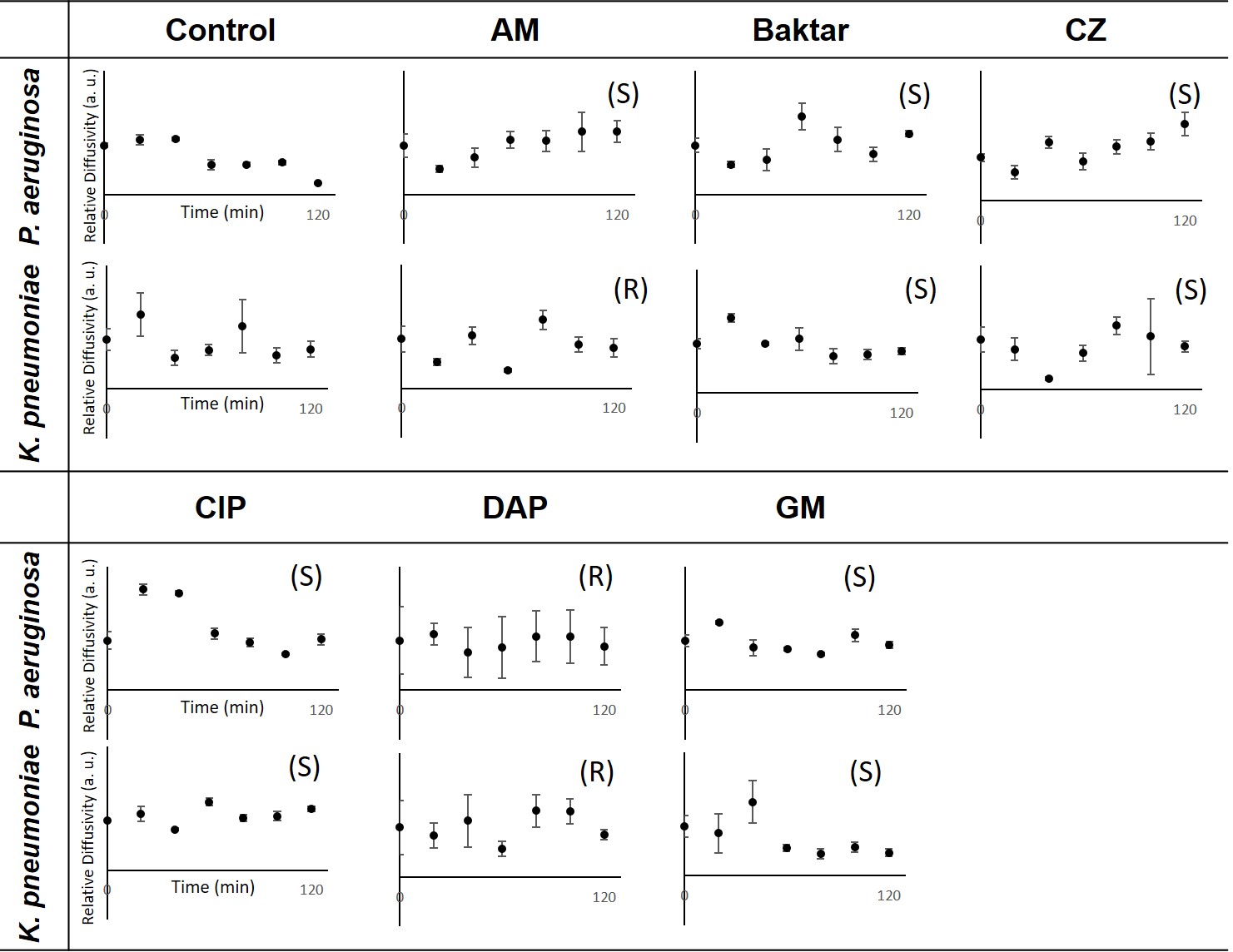


**Figure S7.** Temporal diffusivity changes of *K. pneumoniae* and *P. aeruginosa* treated with six antibiotics in 2 h. The data were measured every 20 min (n=5). “S” means susceptible while “R” means resistant. All antibiotics were tested at the MIC for the respective bacterium.


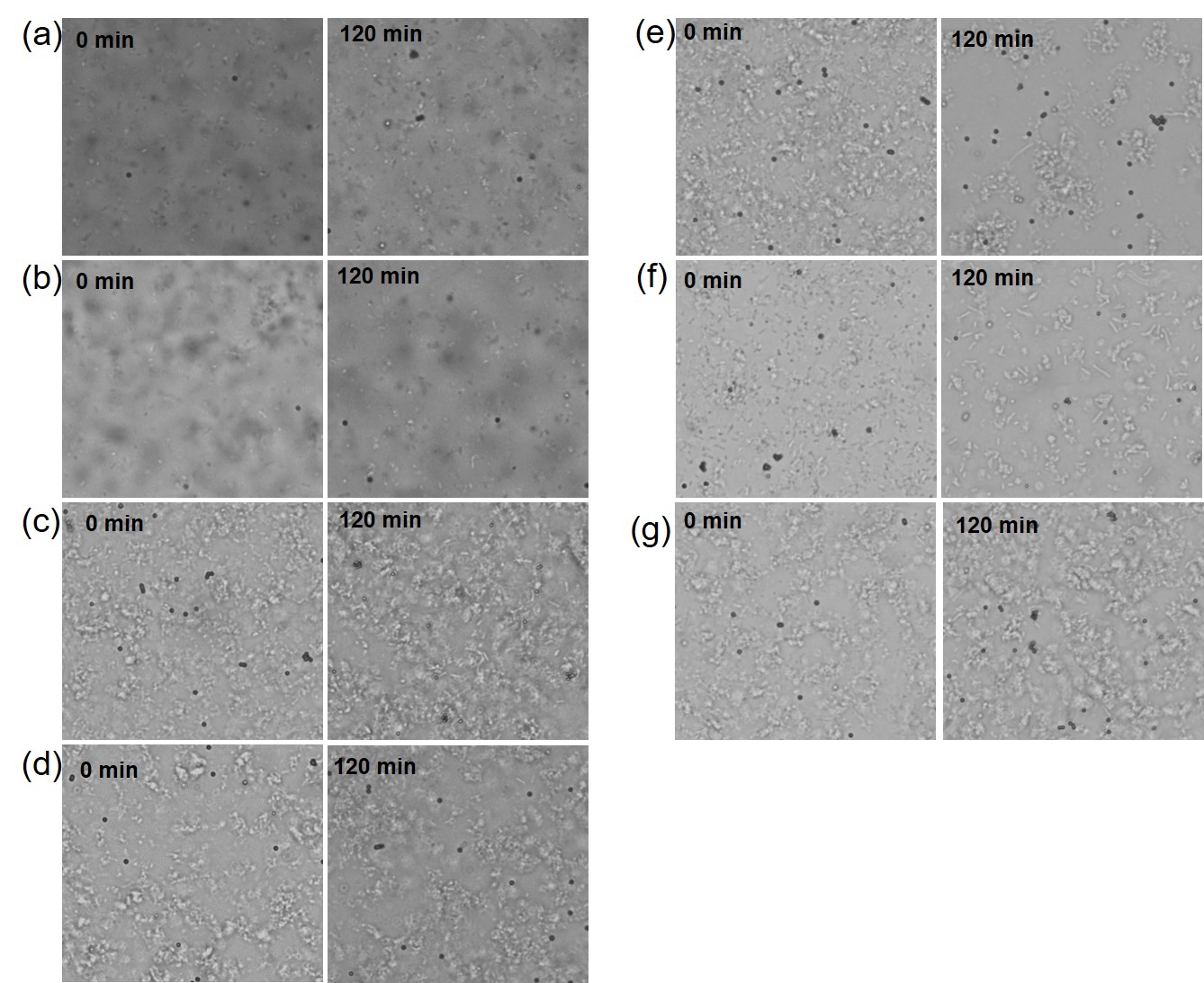


**Figure S8.** Bright-field images of bacterial growth of clinical sample 3 shown in Table 1 at 0 min and 120 min. (a) Control (b) AM (c) Baktar (d) CIP (e) CZ (f) DAP (g) GM.

**Table S1.** Bactericidal mechanisms of the six selected antibiotics and their inhibitory effects on the four bacteria.


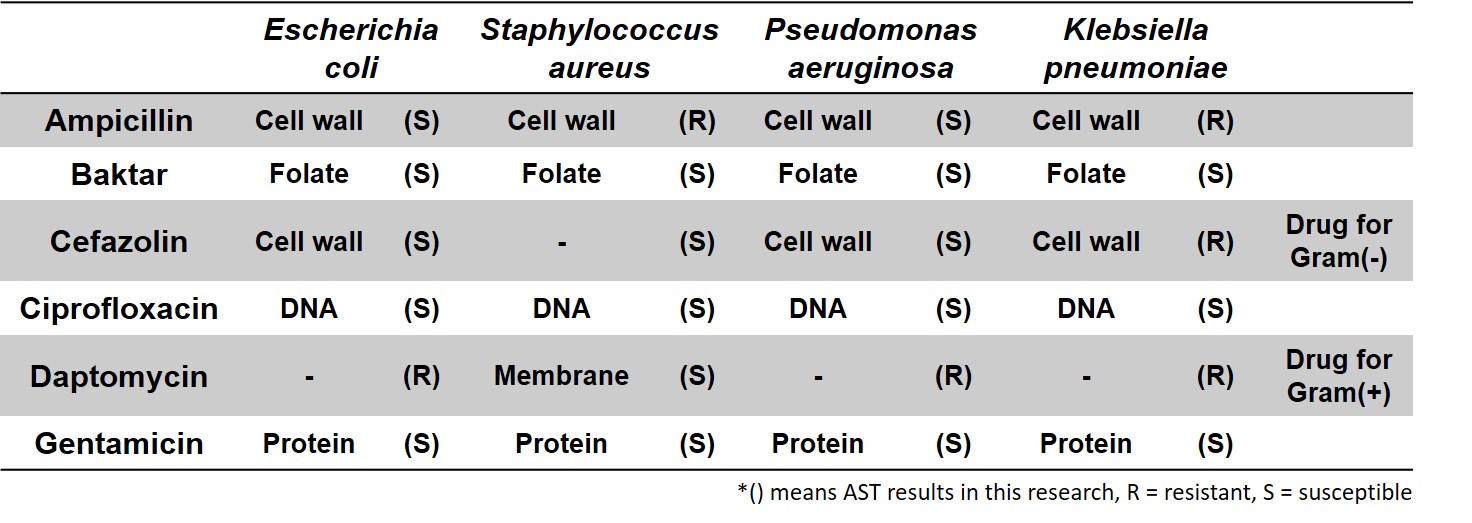


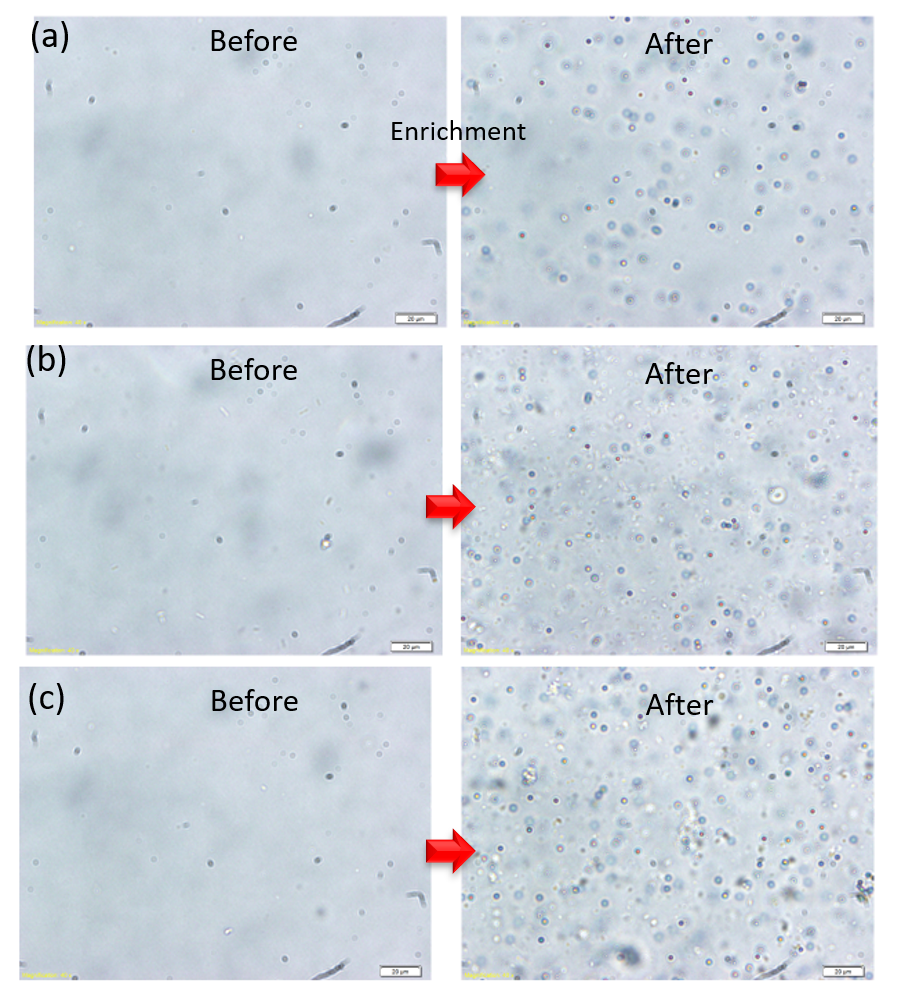


**Figure S9.** Bacterial count enrichment. Three random clinical samples after filtered were concentrated to enrich their bacterial counts. (a) In the first sample, *Proteus miravilis* and *E. coli* were reported and respectively reached concentrations higher than 10^5^ CFU/mL and 10^4^ CGU/mL after enrichment. (b) In the second sample, *Citrobacter koseri* was reported and reached a concentration higher than 10^5^ CFU/mL after enrichment. (c) In the third sample, *Citrobacter koseri* and Corynebacterium striatum were reported and respectively reached concentrations higher than 10^5^ CFU/mL and 10^4^ CGU/mL after enrichment.

***The Single-Blinded test***

The raw data of diffusivuty changes over 2 h of the four clinical samples in the single-blinded test are demonstrated in Figures S10 and S11. The bright field images show proliferative bacteria after 2 h when they are resistant to the antibiotic and vice versa. The slopes of corresponding diffusivity trends in the phase II region are listed next to their curves. Except for th sample 3, all other diffusivity patterns are in good agreements with the Vitek2 and hopital results. The inconsistency was likely arrtibuted to the abnormally high initial baterial count in the sample, which caused disturbance in Bropwnian motion.


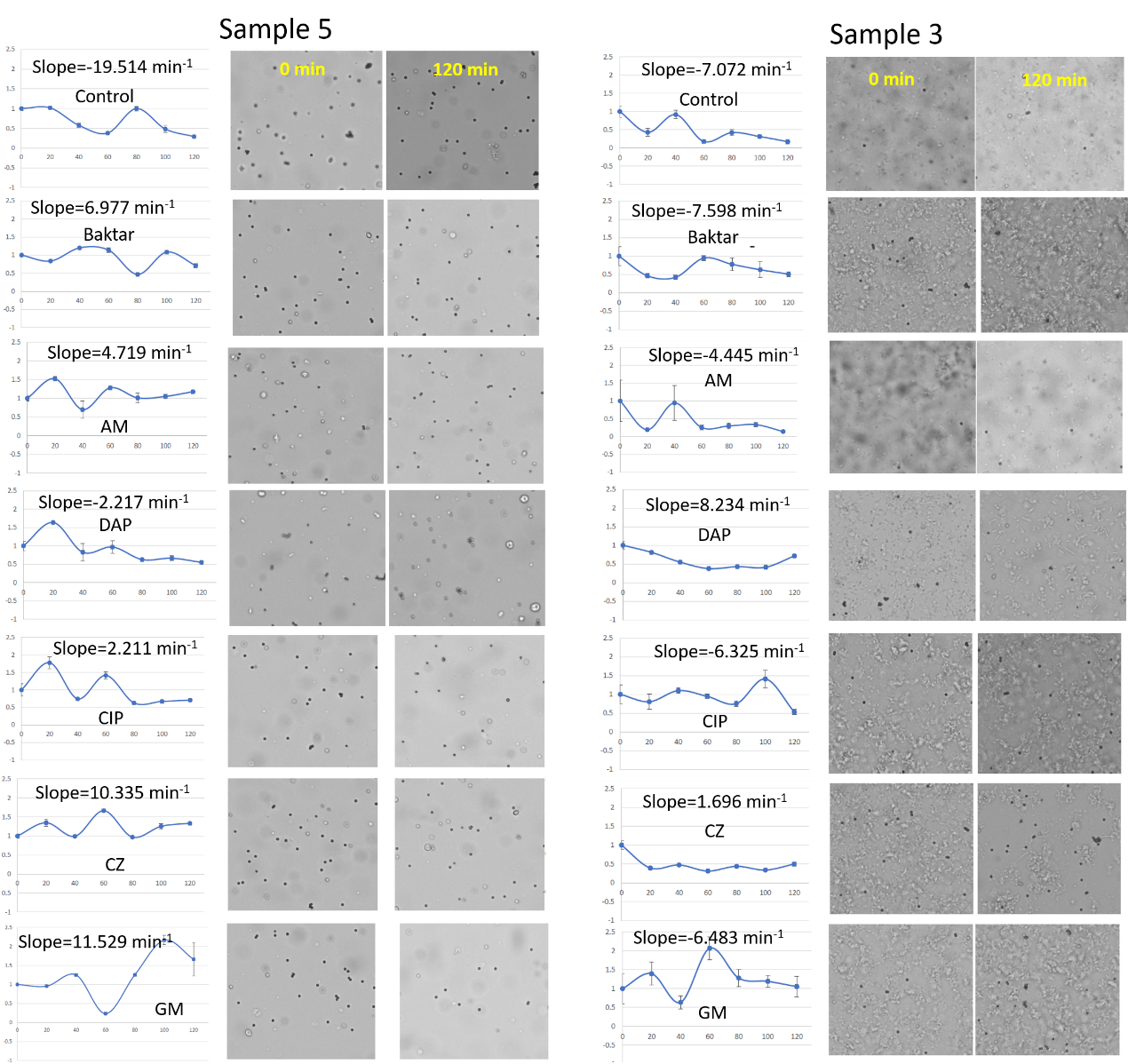


**Figure S10.** Diffusivity patterns and bright-field images recorded at the beginning and after 2-h incubation of samples 3 (right) and 5 (left).


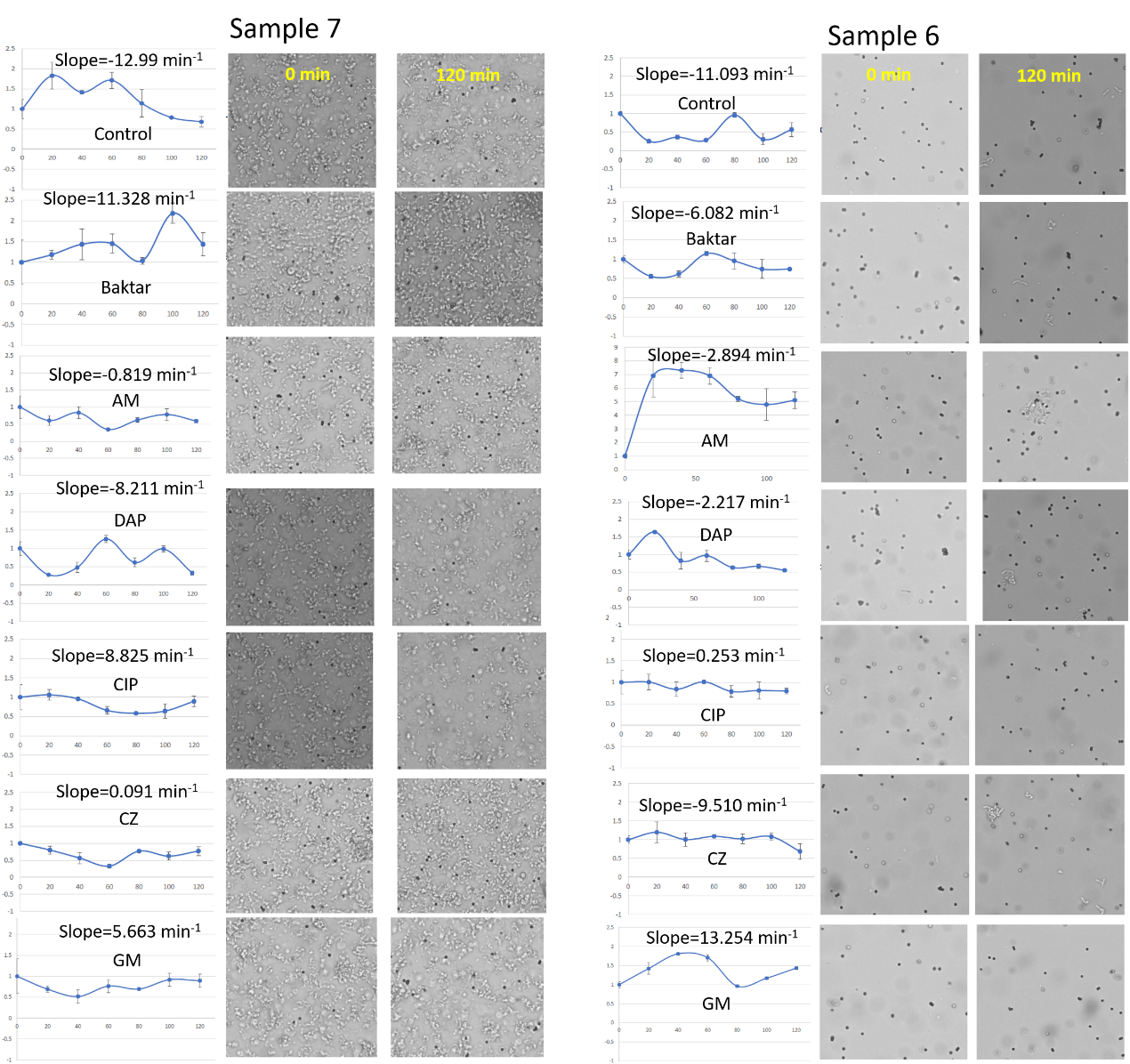


**Figure S11.** Diffusivity patterns and bright-field images recorded at the beginning and after 2-h incubation of samples 6 (right) and 7 (left).

***Determinations of Sensitivity, Specificity, and Accuracy for the Clinical Samples***

In table 1, the data from our optical diffusometry were compared with the Vitek2 results (regarded as standards) and labeled as true positive (resistant in the diffusometry and resistant/intermediate in the Vitek2) and false positive (resistant in the diffusometry but sensitive in the Vitek2) , or true negative (sensitive in the diffusometry and sensitive in the Vitek2) and false negative (sensitive in the diffusometry but resistant/intermediate in the Vitek2). Considering all of the controls were consistent with the Vitek2 counterparts, they were then classified in the true positive group. In addition, the rest samples with no bacteria detected showed level diffusivity changes from the beginning to the end, which implied the same results with the Vitek2. Therefore, they were all classidied in the true negative group. Taking all the above considerations, A table (Table 2) is plotted for the determinations of sensitivity, specificity, and accuracy. The calculations are shown below:

$$Sensitivity=\frac{turue positive}{true positive+false positive}=\frac{13}{14}=92.9\%$$

$$Specificity=\frac{turue negative}{true negative+false negative}=\frac{32}{35}=91.4\%$$

$$Accuracy=\frac{turue positive+true negative}{true positive+false positive+true negative+false negative}=\frac{13+32}{49}=91.8\%$$

**Table S2.** Classifications of positive and negative groups for the 7 clinical results.
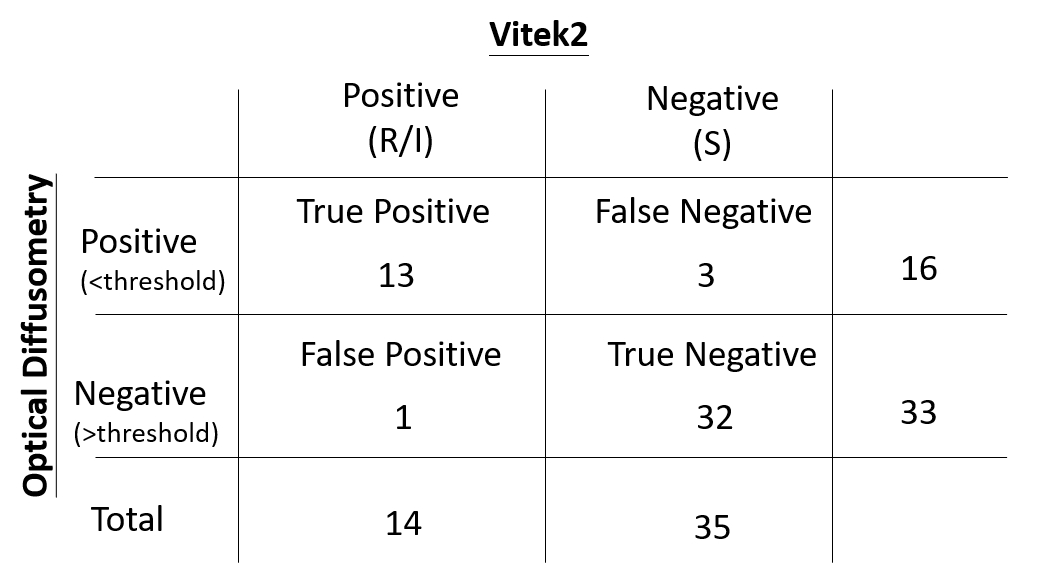

Supplement: Supplementary file 1 [file biosensors-10-00181-s001.zip › Supplementary_v2 (annotated).docx]
